# Transcriptional Response to Deletion of the Phosphatidylserine Decarboxylase Psd1p in the Yeast *Saccharomyces cerevisiae*

**DOI:** 10.1371/journal.pone.0077380

**Published:** 2013-10-11

**Authors:** Martina Gsell, Gerald Mascher, Irmgard Schuiki, Birgit Ploier, Claudia Hrastnik, Günther Daum

**Affiliations:** Institute of Biochemistry, Graz University of Technology, Graz, Austria; Université de Nice-CNRS, France

## Abstract

In the yeast, *Saccharomyces cerevisiae*, the synthesis of the essential phospholipid phosphatidylethanolamine (PE) is accomplished by a network of reactions which comprises four different pathways. The enzyme contributing most to PE formation is the mitochondrial phosphatidylserine decarboxylase 1 (Psd1p) which catalyzes conversion of phosphatidylserine (PS) to PE. To study the genome wide effect of an unbalanced cellular and mitochondrial PE level and in particular the contribution of Psd1p to this depletion we performed a DNA microarray analysis with a ∆*psd1* deletion mutant. This approach revealed that 54 yeast genes were significantly up-regulated in the absence of *PSD1* compared to wild type. Surprisingly, marked down-regulation of genes was not observed. A number of different cellular processes in different subcellular compartments were affected in a ∆*psd1* mutant. Deletion mutants bearing defects in all 54 candidate genes, respectively, were analyzed for their growth phenotype and their phospholipid profile. Only three mutants, namely ∆*gpm2*, ∆*gph1* and ∆*rsb1*, were affected in one of these parameters. The possible link of these mutations to PE deficiency and *PSD1* deletion is discussed.

## Introduction

Phosphatidylethanolamine (PE) is an essential phospholipid in many types of cells from bacteria to man. In the yeast, *Saccharomyces cerevisiae*, depletion of PE which is one of the major cellular and mitochondrial phospholipids causes dysfunction of respiration, defects in the assembly of mitochondrial protein complexes, and loss of mitochondrial DNA [1–3]. Moreover, PE plays a specific role due to its unique biophysical properties as a non-bilayer (hexagonal phase) forming lipid [4]. The biosynthesis of PE comprises a complex network of reactions distributed among different organelles in the cell. Four pathways contribute to PE biosynthesis in yeast, namely (i) decarboxylation of phosphatidylserine (PS) catalyzed by phosphatidylserine decarboxylase 1 (Psd1p) in the inner mitochondrial membrane [5–7]; (ii) decarboxylation of PS by Psd2p in a Golgi/vacuolar compartment [8]; (iii) incorporation of ethanolamine through the CDP-ethanolamine branch of the Kennedy pathway [9] in the endoplasmic reticulum [10,11]; and (iv) synthesis of PE through acylation of lyso-PE catalyzed by the acyl-CoA-dependent acyltransferase Ale1p in the mitochondria-associated membrane (MAM) [12,13]. These four pathways form PE with different efficiency [14]. Psd1p is the major supplier of cellular and mitochondrial PE and represents the major cellular *PSD* activity [8]. Inactivation or deletion of the *PSD1* gene leads to a considerable decrease of PE in total cellular and mitochondrial membranes, especially when yeast cells are grown on non-fermentable carbon sources [1,15]. This finding suggests that marked amounts of PE found in all cellular membranes must be derived from mitochondria. However, mechanisms governing PE distribution within the cell are not well understood.

To obtain a more global insight into the biosynthesis of PE, its regulation and the role of PE in organelle membranes with special emphasis on the biosynthetic capacity of Psd1p, we performed DNA microarray analysis using a ∆*psd1* deletion strain. Here, we show that a relatively small number of genes were affected in such a mutant. The corresponding gene products are involved in various cellular processes including transport, carbohydrate metabolism, stress response and energy metabolism. Moreover, a number of unassigned ORFs were detected during this analysis. In a more detailed analysis, genes and gene products were selected from this screening which either affect growth or lipid metabolism. As a result of this selection, three mutants bearing deletions of *GPM2, GPH1* or *RSB1* were analyzed in some more detail.

## Materials and Methods

### Strains and culture conditions

The wild type yeast strains *Saccharomyces cerevisiae* BY4741 and BY4742 and all deletion mutants in the BY4741 background were obtained from the Euroscarf strain collection (Frankfurt, Germany) (see Table 1). Strains were cultivated on YPD media containing 1% yeast extract, 2% peptone and 2% glucose under aerobic conditions with shaking at 30°C.

**Table 1:** Yeast strains used in this study.

Strain	Genotype	Source
BY4741	MATa *his3*Δ1 *leu2*Δ0 *met15*Δ0 *ura3*Δ0	Euroscarf (Frankfurt, Germany)
BY4742	MATα *his3*Δ1 *leu2*Δ0 *lys2*Δ0 *ura3*Δ0	Euroscarf (Frankfurt, Germany)
BY4742 [pUG35-*GPH1*-GFP]	MATα *his3*Δ1 *leu2*Δ0 *lys2*Δ0 *ura3*Δ0 [pUG35-*GPH1*]	This study
BY4742 [pUG35-*RSB1*-GFP]	MATα *his3*Δ1 *leu2*Δ0 *lys2*Δ0 *ura3*Δ0 [pUG35-*RSB1*]	This study
BY4742 [pUG35-*GPM2*-GFP]	MATα *his3*Δ1 *leu2*Δ0 *lys2*Δ0 *ura3*Δ0 [pUG35-*GPM2*]	This study

All deletion strains used in this study were in the BY4741 background.

Growth tests on fermentable and non-fermentable carbon sources were performed on solid media containing 1% yeast extract, 2% peptone, and 2% agar supplemented with 2% glucose, 2.66% lactate, 2% glycerol or 8 mM sorbitol, respectively. For SDS resistance assays on solid media, 0.05% SDS was added to the media immediately prior to pouring plates. MMLac (minimal medium with lactate) culture plates contain 0.67% yeast nitrogen base without amino acids, 0.073% amino acid mix, 2.66% lactate, adjusted to pH 5.5 with KOH and 2% agar.

The BY4742 wild type strain harboring recombinant plasmids was grown in uracil-free minimal medium (0.67% yeast nitrogen base, 2% glucose and amino acid stock) or in uracil- and methionine-free minimal medium for induction of the MET25-promotor. For growth phenotype analysis cell suspensions of overnight cultures grown in YPD were spotted at dilutions 1, 1/10, 1/100, 1/1000 and 1/10000 on YPD, YPLac, MMLac, YPGlycerol, YPSorbitol and YPD with 0.05% SDS. Incubations were carried out at 30°C.

### Strain constructions

*GPH1*, *GPM2* and *RSB1*, respectively, were cloned into the plasmid pUG35 (provided by J. Hegemann and U. Güldener) with standard genetic methods. pUG35 allows a C-terminal in-frame fusion of the yEGFP3 encoding open reading frame to the gene. The expression of the fusion protein is under control of the MET25-promoter, and under methionine restriction the inserted gene is expressed at high yield. The genes *GPH1*, *GPM2* and *RSB1* (full length form) were amplified from yeast chromosomal DNA of the strain *S. cerevisiae* BY4741 by PCR under standard conditions using the proof reading *Ex Taq* ^TM^-DNA polymerase (Takara). The sequence specific primers used are listed in Table 2 (upper part). Primers for *GPH1* amplification include a *BamH*I or a *Cla*I restriction enzyme recognition sequence, respectively. The *GPM2* PCR-product was cut with *BamH*I and *Hind*III and the *RSB1* PCR-product with *BamH*I and *Sal*I to facilitate the subsequent cloning of the amplified DNA into the specific sites of the pUG35 vector. Genes were sequenced by LGC Genomics (Berlin, Germany). BY4742 was transformed with the cloned vector by the lithium acetate method [16].

**Table 2:** PCR primers used in this study.

Primer	Sequence (5’3’)
GPH1*BamH*If	CGCGGATCCTGAACAATGCCGCCAGCTAGTAC-3´
GPH1*Cla*Ir	CCCATCGATAGTCACTGGTTCAACGTTCCAAATG-3´
RSB1*BamH*If	CGCGGATCCGGTGGTATGGTACCGAACCTTC-3´
RSB1*Sal*Ir	CCCGTCGACAAGTTTAGCCTTCTTTTTAGAGGAAAC-3´
GPM2*BamH*If	CGCGGATCCATGACTGCAAGCACACCATCCAA-3´
GPM2*Hind*IIIr	CCCAAGCTTAGGATTTTTTATGAAACCCTCATTACGG-3´
Act1-RTfwd	CCAGCCTTCTACGTTTCCATCCAAG
Act1-RTrev	GACGTGAGTAACACCATCACCGGA
Psd2_RTfw	GCGCCACAAGATTATCACCGGTTTCACTC
Psd2_RTrev	CTAACTCACTACGAACGGCCATTGGATTTACAG
Ale1_RTfw	CTTGCGAGAAGATGGTGTCACTCCTTTG
Ale1_RTrev	GGTTGCACCATGTAACCAAATGCTAGTTTAATTG
Eki_RTfw	CAATCTAATCATCGAATGGAGGCCTTGTACAC
Eki_RTrev	CATCACCTAGTCCAGCCTTTTCGCTC
Ect1_RTfw	GCCGTTATTATCGATGCTGACGCCAC
Ect1_RTrev	GTCAAGTATTCACTGAATTTTCCGGCAGCAG
Ept_RTfw	CTCTTTCATCCTTTCCGTTGGTTTCACGGGAG
Ept_RTrev	CATGGGTGCATTGAACATGGGAAAGCTC
Dpl1_RTfw	GTCGTGCCAAGAAATAGTCGGTGCAGCAATG
Dpl1_RTrev	GACTGAATATCTAGGGTTGCCCATTATATTCAGGTCTG
Gph1_RTfw	CCACGCAAGGTTTCAATCTTTGGTGGTAAGAG
Gph1_RTrev	CGTCGTTATTAACAATGTCAGCAACACAGTTGATC
Gpm2_RTfw	GTGGGCCATGGAAGTTCAGTGAGATC
Gpm2_RTrev	CAACGACTAAGGGGATACCATTTGGAATATCAAC
Rsb1_RTfw	GATGGCCATCGCTACGGTCACTTTG
Rsb1_RTrev	GTAAAGTTTCAGGATCAACATGGCCCGGTG

Upper part: PCR primers used for the amplification of genes. Recognition sites for restriction enzymes are underlined. Lower part: Primers used for qRT-PCR.

### RNA isolation and Real Time PCR

For the isolation of total RNA cells were grown to an OD_600_ of 5 on YPD at 30°C. RNA was isolated using the RNeasy kit from Qiagen as described by the manufacturer. After DNaseI digestion, Real Time PCR was performed using SuperScript III Platinum SYBR Green One-Step qRT-PCR Kit (Invitrogen) by following the manufacturer’s instructions. Amplification was measured using an ABI 7500 instrument (Applied Biosystems). Samples were evaluated using the ∆∆Ct method described by Livak and Schmittgen [17]. Differences in mRNA expression were calculated after *ACT1* normalization relative to the control. Primers used for Real Time-PCR are listed in Table 2 (lower part)

### Isolation of plasma membrane and mitochondria

Yeast cells were grown aerobically in YPD to the early stationary phase at 30°C. Then, cells were disrupted with glass beads using a Merckenschlager homogenizer under CO_2_-cooling. Cell extracts were cleared of glass beads, unbroken cells and cell debris by centrifugation at 2,500 x g for 5 min. The supernatant fraction represented the homogenate. Crude plasma membrane was isolated essentially as described by Serrano [18] and further purified as reported by van den Hazel et al. [19] and Pichler et al. [20].

To isolate mitochondria, spheroplasts were prepared and homogenized in breaking buffer consisting of 0.6 M mannitol, 10 mM Tris, pH 7.4 and 1 mM PMSF by using a Dounce homogenizer as described previously [21]. Unbroken cells and debris were removed by centrifugation at 3,000 x g for 5 min. The resulting supernatant was used to isolate mitochondria by published procedures [22].

Relative enrichment of markers and cross-contamination of subcellular fractions were assessed as described by Zinser and Daum [23]. Proteins were quantified by the method of Lowry et al. [24] using bovine serum albumin (BSA) as standard. SDS-PAGE was carried out as published by Laemmli [25]. Western blot analysis of proteins from subcellular fractions was performed as described by Haid and Suissa [26]. Immunoreactive bands were visualized by enzyme-linked immunosorbent assay using a peroxide-linked secondary antibody (Sigma-Aldrich, St Louis, MO) following the manufacturer´s instructions. 

### Microarray analysis

For the preparation of RNA, BY4741 and Δ*psd1* were pre-grown overnight. For main cultures, inoculations to an OD_600_ of 0.1 in fresh medium were made by diluting pre-cultures. Cells were grown to the logarithmic phase and harvested by centrifugation. Cell pellets were shock frozen in liquid nitrogen and stored at -70°C till use. Total RNA was isolated using an RNeasy Kit (Qiagen) including DNase I treatment according to the manufacturer´s instructions. Yeast lysates were prepared by mechanical disruption of cells using a bead mill. Integrity of RNA was tested by agarose gel electrophoresis and determination of the 260 to 280 nm absorbance ratio. The RNA concentration was estimated by measuring the absorbance at 260 nm.

For expression profiling, 1 µg of total RNA was linearly amplified and biotinylated using the One-Cycle Target Labeling Kit (Affymetrix, Santa Clara, CA) according to the manufacturer’s instructions. 15 µg of labeled and fragmented cRNA were hybridized to Affymetrix Yeast 2.0 Gene Chip® arrays (Affymetrix). After hybridization, the arrays were washed and stained in a Fluidics Station 450 (Affymetrix) with the recommended washing procedure. Biotinylated cRNA bound to target molecules was detected with streptavidin-coupled phycoerithrin, biotinylated anti-streptavidin IgG antibodies and again streptavidin-coupled phycoerithrin according to the manufacturer’s protocol. Arrays were scanned using the GCS3000 Gene Chip scanner (Affymetrix) and GCOS 1.4 software. Scanned images were subjected to visual inspection to test for hybridization artifacts and proper grid alignment, and analyzed with Microarray Suite 5.0 (Affymetrix) to generate report files for quality control. 

Statistical data analysis was performed using the bioconductor packages “affy” and “limma” [27,28]. Initially, the expression data from all arrays were normalized with RMA [29] to yield log_2_-transformed signal values. The assay was performed in two independent experiments with two and four samples, respectively. A batch effect was observed (data not shown). A linear model was generated including the factors “batch” and “strain” to correct for this effect. The signal values were then averaged for the individual subgroups and differences in the expression level were calculated as x-fold change. Differences between Δ*psd1* and BY4741 strains were extracted and analyzed with the moderated T-test (empirical Bayes method). Transcripts with an at least two-fold change of expression level and a p-value of less than 0.05 were regarded as differentially expressed. Functions of differentially expressed transcripts were annotated using the latest array annotation in the NetAFFX analysis center (www.affymetrix.com/analysis/index.affx). 

### Bioinformatic analysis

To retrieve information about ORFs of interest, the Saccharomyces Genome Database (SGD, http://www.yeastgenome.org/) was queried. The SGD tool GO Slim Mapper [28] was used to assign the general Gene Ontology (GO) terms to identified ORFs. Genes were categorized by using the SGD GO Slim Mapper (http://db.yeastgenome.org/cgi-bin/GO/goTermMapper) to define the biological processes, molecular functions and cellular components of gene products, in conjunction with GO annotations for yeast gene products curated by the SGD. 

### Phospholipid analysis

Phospholipids were extracted from the homogenate containing 3 mg protein or from mitochondria and plasma membrane fractions containing 2 mg protein, respectively, by the procedure of Folch et al. [30] using 3 ml chloroform/methanol (2:1, v/v). Individual phospholipids were separated by two-dimensional thin-layer chromatography (TLC) on silica gel 60 plates (Merck, Darmstadt, Germany) using chloroform/methanol/25% NH_3_ (50:25:6; per vol.) as first, and chloroform/acetone/methanol/acetic acid/water (50:20:10:10:5; per vol.) as second developing solvents. Lipids were stained with iodine vapor, scraped off the plate, and quantified by the method of Broekhuyse [31].

### Fluorescence microscopy

Fluorescence microscopy was performed to localize the C-terminally GFP-tagged proteins Gph1p, Rsb1p and Gpm2p within the cell. The yeast strains carrying fusion proteins were grown at 30°C in uracil- and methionine-free minimal medium. At different time points samples were taken. Fluorescence microscopic analysis was performed using a Zeiss Axiovert 35 microscope with a 100-fold oil immersion objective, a UV lamp and a detection range between 450 and 490 nm. Images were taken with a CCD camera.

## Results

### Influence of PSD1 deletion on gene expression in *Saccharomyces cerevisiae*

The present study was aimed at identifying genes of the yeast *Saccharomyces cerevisiae* whose expression levels were changed in response to PE depletion and deletion of the major producer of PE in the cell, the mitochondrial PS decarboxylase Psd1p. The genome-wide expression pattern was analyzed using cDNA microarrays (Affymetrix gene chip system). Among 5,841 open reading frames (transcripts) examined, 54 genes were significantly up-regulated in a *∆psd1* deletion strain. Significant down-regulation of genes was not observed under these conditions. The list of genes and their expression levels (x-fold change values) are shown in the Table S1. The logFC (log *fold change*) values were in the range from -6.09 (*PSD1*) to +2.72 (*DAN1*). All 54 up-regulated ORFs encode for non-essential proteins. 

### Analysis of ORFs affected by PSD1 deletion

ORFs detected by the above mentioned assay were bioinformatically analyzed using SGD GO slim mapping [29]. This bioinformatic tool sorted the 54 ORFs according to their contribution to biological processes, molecular function and subcellular localization (Tables 3-5). Among the up-regulated genes a large fraction was related to transport, carbohydrate metabolism, generation of precursor metabolites and energy, and response to stress (Table 3). 23 ORFs with unknown biological process were also up-regulated under PE limitation. As molecular function, 18 ORFs were related to catalytic enzyme activities, namely hydrolase, transferase, oxidoreductase, phosphatase and isomerase activity (Table 4). Five ORFs were assigned to transporter activity. Other molecular functions of genes identified were lipid binding, RNA binding, protein binding, transcription regulator activity, signal transducer activity, DNA binding and enzyme regulator activity. The molecular function of 28 ORFs is unknown. The largest group of gene products was localized to the cytoplasm, followed by the nucleus, plasma membrane, cell wall and mitochondria (Table 5). In addition, some ORFs were assigned to the vacuole, endoplasmic reticulum, cytoplasmic-membrane bound vesicles, ribosomes and lipid droplets. Subcellular localization of 14 gene products is unknown. 

**Table 3:** GO slim terms Biological Process of the set of up-regulated genes in the absence of *PSD1*.

**Biological Process**	**Genes**
Biological process unknown	*BDH2, FMP23, PAU3, GPM2, UGX2, PAU5, YGR066C, SET4, YJR005C-A, YKL071W, PAU16, YLR149C, TMA10, YMR317W, PAU19, YTP1, TIR4, YOR289W, FRE5, PAU21, PAU22, PAU15, YLR346C*
Transport	*PDR15, FIT1, HXK1, ARN1, ARN2, HXT5, DAN1, RSB1, HES1, FIT2, FIT3, RTC2*
Carbohydrate metabolic process	*GLC3, HXK1, AMS1, SOL4, TDH1, TSL1, PGM2, GPH1*
Cellular homeostasis	*ARN1, ARN2, TIS11 ,PGM2, FIT1, FIT2, FIT3*
Generation of precursor metabolites and energy	*GLC3, HXK1, TDH1, PGM2, GPH1*
Response to stress	*HSP26, HSP12, XBP1, TSL1, DDR2*
Cofactor metabolic process	*SOL4, ARN2, GTO3*
Membrane organization and biogenesis	*RSB1, HES1, HSP12*
RNA metabolic process	*RTC3, TIS11*
Response to chemical stimulus	*PDR15, HSP12*
Protein folding	*HSP26*
Cell wall organization and biogenesis	*ECM4*
Vitamin metabolic process	*SOL4*
Signal transduction	*GPG1*
Sporulation	*YNL194C*
Vesicle-mediated transport	*HES1*
Anatomical structure morphogenesis	*HES1*
Nuclear organization and biogenesis	*GSP2*
Response to starvation	*PHM8*

**Table 4:** GO slim terms Molecular Function of the set of up-regulated genes in the absence of *PSD1*.

**Molecular Function**	**Genes**
Molecular function unknown	*FMP23, PAU3, GPM2, UGX2, FIT1, PAU5, YGR066C, RTC3, PAU15, SET4, YJR005C-A, DAN1, YKL071W, PAU16, YLR149C, TMA10, YLR346C, YMR317W, PAU19, YNL194C, YTP1, DDR2, TIR4, YOR289W, FIT2, FIT3, PAU21, PAU22*
Hydrolase activity	*PDR15, AMS1, SOL4, TSL1, RSB1, GSP2*
Transferase activity	*GLC3, HXK1, ECM4, TSL1, GTO3, GPH1*
Transporter activity	*PDR15, ARN1, ARN2, HXT5, RTC2*
Oxidoreductase activity	*BDH2, TDH1, FRE5*
Lipid binding	*HES1, HSP12*
RNA binding	*TIS11, HSP26*
Phosphatase activity	*PHM8, TSL1*
Isomerase activity	*PGM2*
Protein binding	*HSP26*
Transcription regulator activity	*XBP1*
Signal transducer activity	*GPG1*
DNA binding	*XBP1*
Enzyme regulator activity	*TSL1*

**Table 5:** GO slim terms Cellular Component of the set of up-regulated genes in the absence of *PSD1*.

**Cellular Component**	**Genes**
Cytoplasm	*BDH2, FMP23, HSP26, RTC2, GPM2, GLC3, PHM8, HSP12, HXK1, AMS1, SOL4, ARN1, ARN2, RTC3, TDH1, YKL071W, ECM4, TIS11, TMA10, YLR346C, TSL1, PGM2, GTO3, YNL194C ,DDR2, RSB1, YOR289W, FRE5, GPH1, PAU5*
Cellular component unknown	*PAU3, UGX2, GPG1, YGR066C, PAU15, SET4, YJR005C-A, PAU16, YLR149C, YMR317W, PAU19, HES1, PAU21, PAU22*
Nucleus	*BDH2, HSP26, PHM8, HSP12, SOL4, RTC3, XBP1, TIS11, TMA10, GSP2, YOR289W*
Plasma membrane	*HSP12, ARN2, HXT5, RSB1, ARN1, TDH1, YNL194C*
Cell wall	*FIT1, TDH1, DAN1, TIR4, FIT2, FIT3*
Mitochondrion	*FMP23, RTC2, TDH1, YLR346C, FRE5*
Vacuole	*AMS1 ,DDR2, RTC2*
Endoplasmic reticulum	*YNL194C, RSB1*
Cytoplasmic membrane-bounded vesicle	*ARN1, ARN2*
Ribosome	*TMA10*
Lipid droplet	*TDH1*

Taken together, the bioinformatic analysis described above showed that depletion of PE and deletion of *PSD1* in yeast cells appear to affect a number of biological processes. The fact that all genes were up-regulated upon deletion of *PSD1* suggests compensatory gain-of-function. Such a response appears logical for organelle membrane associated gene products (see Table 5) whose activity may at least in part depend on the presence of PE in the membrane environment. Various enzymes and transport related proteins (see Table 4) might be affected in that way. How PE levels in the cell may affect the activity of cytosolic proteins is less obvious. In these cases, secondary effects may be regarded as the reason for this finding. Interestingly, a number of up-regulated genes belong to the so-called seripauperin multigene family (*PAU3, PAU15, PAU16, PAU19, PAU21* and *PAU22*). These gene products are mostly located in the subtelomeric regions of chromosomes [32]. Although the exact role of *PAU* genes and their gene products is still unclear, they are considered to be induced by stress and anaerobiosis [33]. *PAU* genes are extremely homologous to each other and also share homology with Tir and Dan proteins. *TIR4* and *DAN1* were also overexpressed in a ∆*psd1* deletion mutant. Tir and Dan proteins are also induced by anaerobiosis [34,35]. Due to the large number of protein family members most likely with overlapping functions single mutations of these genes do not lead to obvious phenotypes. The link of *PAU*, *TIR* and *DAN* genes to *PSD1* may reflect the importance of Psd1p for respiratory function. Recent work by Böttinger et al. [36] showed that respiration is compromised in a ∆*psd1* mutant. Overexpression of Pau, Tir and Dan proteins might be a compensatory reaction on this defect.

**Figure 1:**
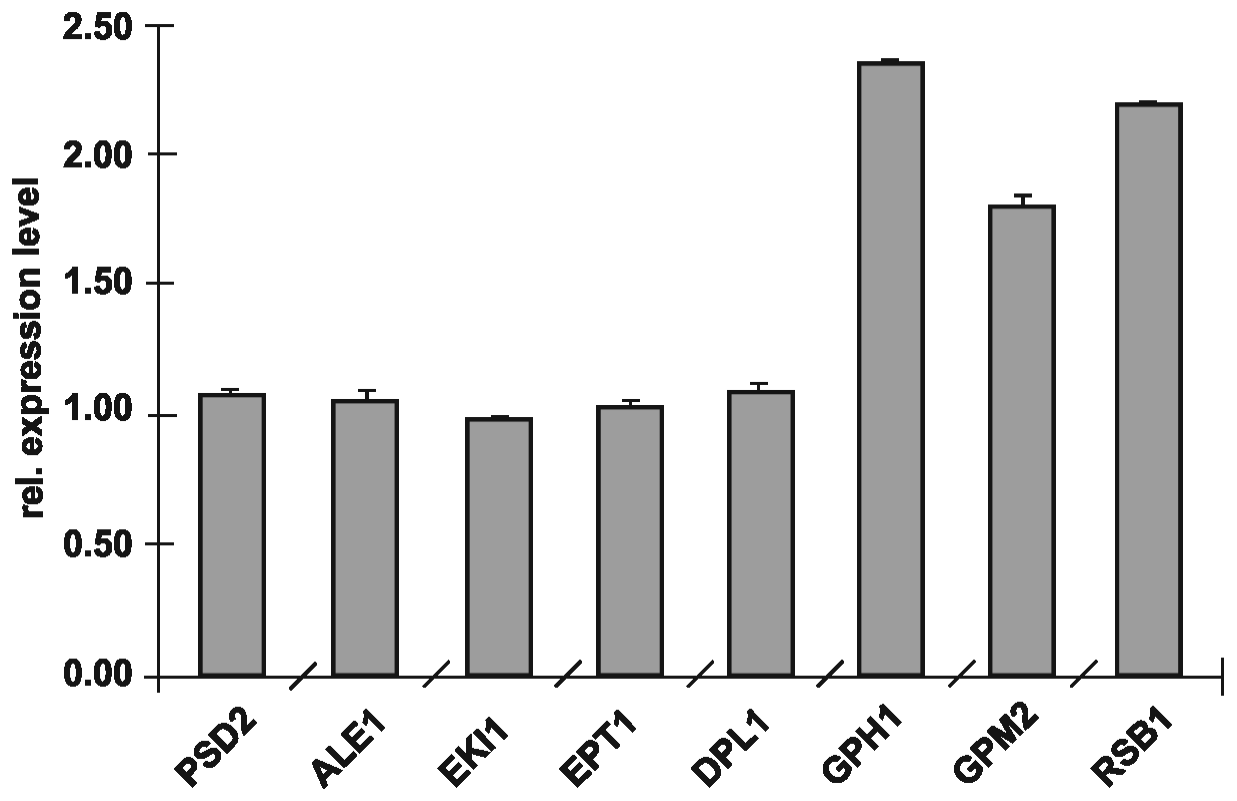
Gene expression analysis. Relative gene expression of *PSD2*, *ALE1*, *EKI1*, *EPT1*, *DPL1*, *GPH1*, *GPM2* and *RSB1* was measured by qRT-PCR from isolated RNA of wild type and *Δpsd1*. Expression of the respective genes in wild type was set at 1 and values obtained with RNA isolated from *Δpsd1* were set in relation. Data are mean values from three independent experiments with the respective deviation.

Surprisingly, genes encoding enzymes of the other three PE biosynthetic pathways were not up-regulated in the ∆*psd1* mutant to compensate eventually for PE depletion. *PSD2, ALE1* and genes of the Kennedy pathway, namely *EKI1, EPT1* and *DPL1* were additionally tested with qRT-PCR to confirm the DNA microarray results. Indeed no changes in the gene expression levels compared to wild type were observed (Figure 1). This analysis also confirmed the increased transcription levels of *GPH1*, *GPM2* and *RSB1* in the individual tests.

### Phenotype analysis of deletion mutants compromised in genes up-regulated in ∆psd1

**Figure 2:**
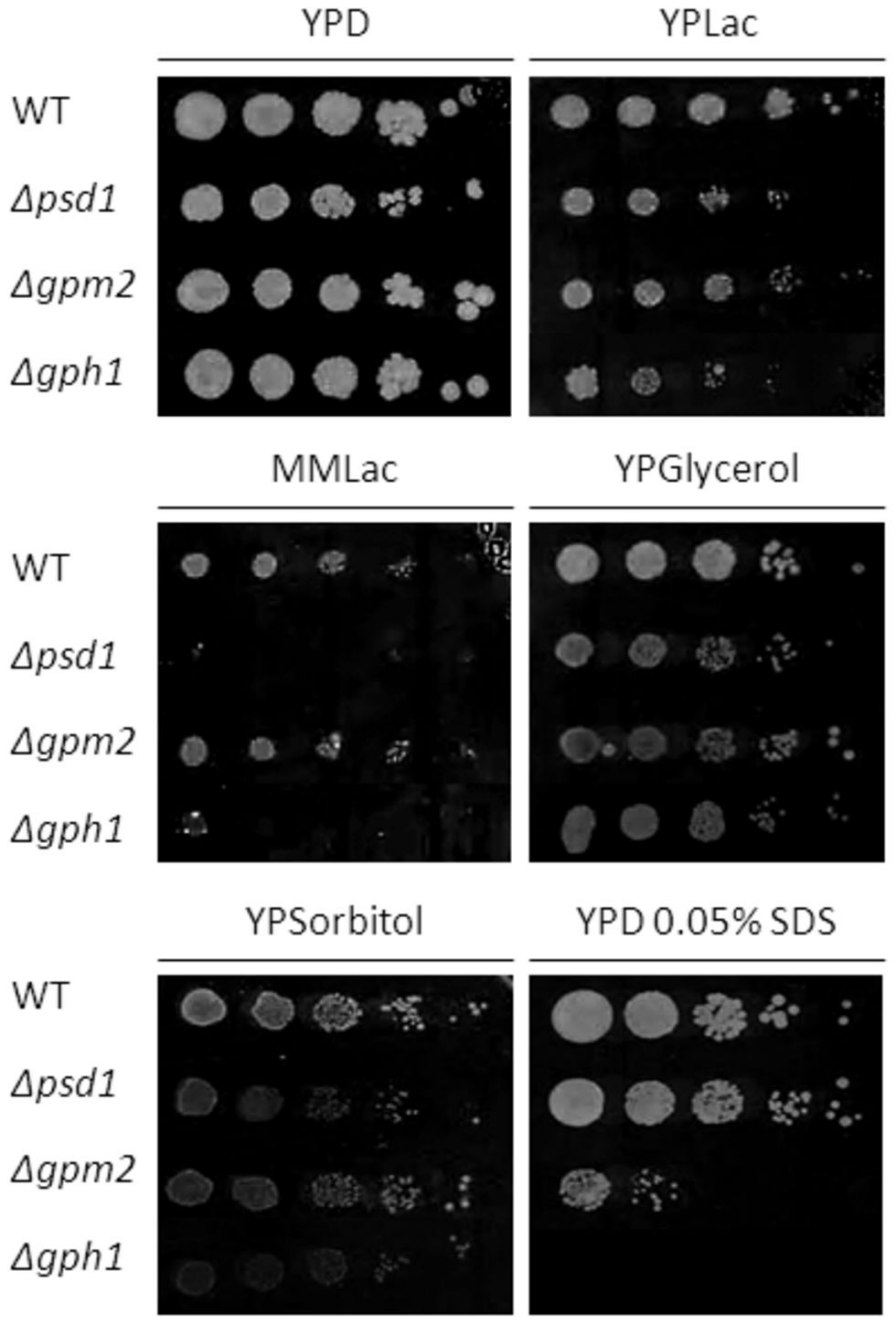
Growth analysis of yeast strains deleted of genes up-regulated in the absence of *PSD1*. Strains as indicated were grown on YPD, YPLac, MMLac, YPGlycerol, YPSorbitol, and YPD with 0.05% SDS. Cell suspensions of strains listed were spotted at dilutions 1, 1/10, 1/100, 1/1000, and 1/10000. Incubation was carried out at 30°C.

Mutants deleted of genes which were found to be up-regulated in *∆psd1* were tested for their growth phenotype on different media. Fermentable and non-fermentable carbon sources were used for these tests. It has to be noted that on non-fermentable carbon sources like lactate and glycerol the requirement for PE increases due to intense proliferation of mitochondria and the importance of PE for cell respiration [1]. Under these conditions even a single deletion of *PSD1* leads to a growth defect (Figure 2). Additionally, osmotic stability of strains on sorbitol and SDS sensitivity was analyzed. Surprisingly, most of the 54 mutants tested did not show changes in their growth behavior compared to wild type (data not shown). Only ∆*gpm2* and ∆*gph1* deletion mutants exhibited growth defects on certain media. Whereas ∆*gpm2* grew on YPD with glucose as carbon source like wild type, a slight growth defect was observed on non-fermentable carbon sources, suggesting a respiratory defect in these cells. A strong growth defect of ∆*gpm2* was observed in the presence of SDS indicating decreased stability of the plasma membrane and/or the cell wall. Gph1p seems to play also a role in respiration and cell wall/plasma membrane organization because of the strongly reduced growth of the deletion mutant on lactate and SDS containing media. The ∆*gph1* deletion mutant also exhibits osmotic instability on sorbitol. These mutant strains showing defects in osmotic stability were also tested for oxidative stress in the presence of H_2_O_2_. There was, however, no significant difference of growth of the mutants compared to the wild type or the ∆*psd1* deletion mutant, respectively (data not shown). Double deletions of ∆*gpm2*, ∆*gph1* and *∆rsb1*, respectively, with *∆psd1* were also tested. A *Δgph1Δpsd1* double mutant was successfully constructed indicating that there was no synthetic lethality of Δ*gph1* and Δ*psd1*. On the other hand, the construction of *Δgpm2Δpsd1* and *Δrsb1Δpsd1* double mutants failed indicating synthetic lethality of the respective pairs of genes thus reinforcing the possible connection in lipid homeostasis.

### Phospholipid analysis of deletion mutants compromised in genes up-regulated in ∆psd1

To estimate the possible involvement of the up-regulated genes in phospholipid metabolism we established phospholipid profiles of all deletion mutants. In wild type homogenate, the major phospholipids were phosphatidylcholine (PC), PE and phosphatidylinositol (PI) (Table 6). Lysophospholipids (LPL), phosphatidic acid (PA), cardiolipin (CL) and dimethylphosphatidylethanolamine (DMPE) were present only at minor amounts. As shown before [2,3,15,37,38] deletion of *PSD1* causes a depletion of PE in the cell homogenate and mitochondria compared to wild type. Interestingly, no major changes in the PE level were observed in total cell extracts of mutants detected of genes which were up-regulated in the absence of *PSD1*. Only in a strain deleted of *RSB1* a slight decrease of PE was measured which was accompanied by an increase in PI, PS and PA (see Table 6). However, changes in the phospholipid pattern were also detected in Δ*gpm2* and Δ*gph1*. In Δ*gpm2* these changes were moderate, but in Δ*gph1* markedly increased levels of PI, DMPE and PA mainly at the expense of PC were detected.

**Table 6:** Phospholipid composition of cell-free homogenate, mitochondria and plasma membrane from cells grown on YPD.

		Phospholipids in cell-free homogenate, mitochondria and plasma membrane (mol %)							
CF	Strain	LPL	PI	PS	PC	PE	CL	DMPE	PA
Homogenate	WT	1.52 ± 0.21	9.93 ± 3.53	8.75 ± 0.51	45.08 ± 1.82	26.61 ± 2.53	3.35 ± 0.29	4.43 ± 0.70	0.71 ± 0.38
	Δ*psd1*	2.15 ± 0.76	16.00 ± 1.17	11.28 ± 2.38	46.68 ± 2.34	18.23 ± 1.23	1.50 ± 0.64	2.75 ± 0.44	0.95 ± 0.23
	Δ*gpm2*	1.03 ± 0.64	13.07 ± 5.34	7.73 ± 1.83	46.63 ± 3.92	23.40 ± 2.46	3.94 ± 0.84	2.75 ± 1.19	0.90 ± 0.50
	Δ*gph1*	0.62 ± 0.33	14.04 ± 0.74	9.18 ± 0.85	39.30 ± 1.01	25.66 ± 0.47	2.13 ± 0.37	6.85 ± 0.52	1.73 ± 1.30
	Δ*rsb1*	1.04 ± 0.13	17.16 ± 1.14	10.50 ± 1.03	40.36 ± 0.08	22.64 ± 0.91	2.84 ± 0.50	3.51 ± 0.62	1.76 ± 0.62
Mitochondria	WT	1.92 ± 1.11	8.06 ± 1.72	4.07 ± 0.60	40.65 ± 2.38	30.35 ± 1.36	4.97 ± 3.64	6.58 ± 2.54	2.38 ± 0.60
	Δ*psd1*	2.12 ± 0.46	8.80 ± 3.67	7.31 ± 1.54	46.98 ± 5.42	23.86 ± 3.96	2.87 ± 1.77	3.23 ± 1.56	4.78 ± 2.21
	Δ*gpm2*	1.53 ± 0.44	10.79 ± 2.45	3.68 ± 1.02	45.14 ± 2.27	26.98 ± 1.13	7.34 ± 1.79	1.56 ± 0.35	2.73 ± 0.62
	Δ*gph1*	1.58 ± 0.68	9.49 ± 2.30	6.94 ± 1.00	35.90 ± 2.32	33.94 ± 0.38	6.17 ± 1.51	3.66 ± 0.26	2.10 ± 0.58
	Δ*rsb1*	1.12 ± 0.11	13.33 ± 0.52	5.29 ± 0.44	34.86 ± 1.24	29.15 ± 1.13	8.18 ± 0.26	5.70 ± 0.25	2.05 ± 0.20
Plasma membrane	WT	2.26 ± 0.96	12.38 ± 2.12	26.18 ± 2.79	18.18 ± 1.38	32.06 ± 4.14	0.69 ± 0.10	2.23 ± 0.43	5.76 ± 0.61
	Δ*psd1*	2.80 ± 0.53	17.11 ± 4.11	27.27 ± 3.20	21.27 ± 3.32	23.47 ± 3.61	0.64 ± 0.32	1.90 ± 0.30	5.30 ± 2.18
	Δ*gpm2*	2.19 ± 0.70	12.90 ± 1.71	24.31 ± 3.02	21.36 ± 1.93	30.94 ± 3.25	0.73 ± 0.34	1.91 ± 0.64	5.42 ± 1.26
	Δ*gph1*	1.88 ± 0.96	14.82 ± 2.93	24.90 ± 3.96	11.04 ± 2.25	36.27 ± 2.77	0.58 ± 0.27	2.44 ± 0.55	7.84 ± 3.13
	Δ*rsb1*	1.49 ± 0.40	13.63 ± 0.44	24.74 ± 2.98	20.17 ± 1.44	31.47 ± 2.71	0.12 ± 0.17	2.21 ± 0.62	5.72 ± 0.60

CF, cellular fraction; LPL, lysophospholipids; PI, phosphatidylinositol; PS, phosphatidylserine; PE, phosphatidylethanolamine; PC, phosphatidylcholine; CL, cardiolipin; DMPE, dimethylphosphatidylethanolamine; PA, phosphatidic acid. Mean values of at least three measurements and standard deviations are shown.

To address possible effects of the lipid profile on respiration and osmotic stability as suggested by the growth phenotype analysis (see Figure 2) we analyzed isolated mitochondrial and plasma membrane fractions of the respective strains. In the ∆*gpm2* mutant, a 75% reduction of the DMPE level in mitochondria was observed resembling the effect in ∆*psd1*. DMPE is an intermediate product in the methylation pathway from PE to PC catalyzed by Cho2p and Opi3p. Surprisingly, PC levels in the homogenate and especially in mitochondria of ∆*gpm2* were increased over the wild type suggesting that production of PC through the alternative biosynthetic route, the so-called Kennedy pathway, was enhanced. As another marked feature of the ∆*gpm2* strain the CL level in mitochondria was increased. No significant changes were observed in the phospholipid pattern of the plasma membrane. 

Major changes in mitochondria of the ∆*gph1* mutant were reduction of the PC and DMPE levels and an increase of PI and CL. The reduction of PC was even more pronounced in the plasma membrane, where it was reduced to 60% of wild type. The decrease in the PC level was compensated by increased amounts of PE, PI and PA. Deletion of the *RSB1* gene caused a dramatic increase in PI in the homogenate over the wild type at the expense of PC and PE. Changes in PI and PC levels were also detected in mitochondria, whereas the plasma membrane was not affected.

**Figure 3:**
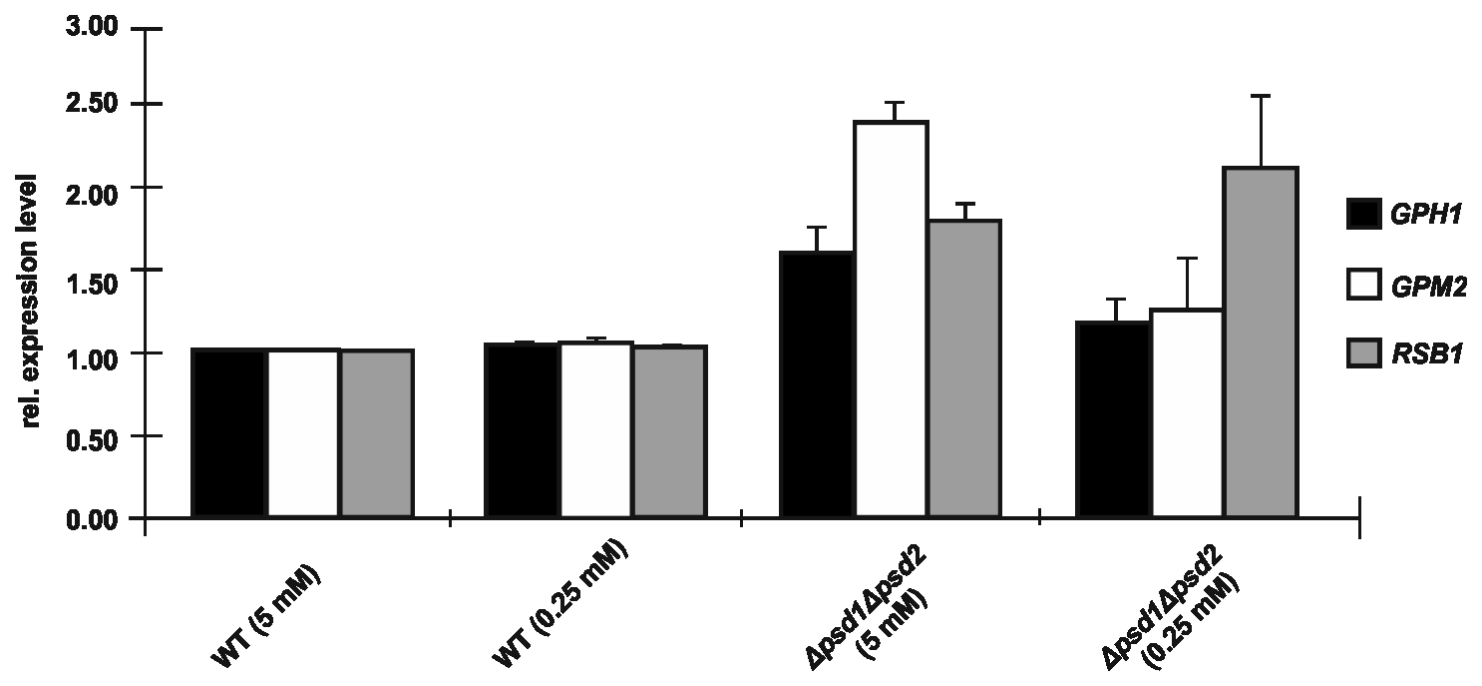
Gene expression of *GPH1*, *GPM2* and *RSB1* in *Δpsd1Δpsd2* with variable supplementation of ethanolamine. Relative gene expression of *GPH1*, *GPM2* and *RSB1* was measured by qRT-PCR from isolated RNA of wild type and *Δpsd1Δpsd2* cultivated in the presence of different amounts of ethanolamine (values in brackets). Expression of the respective genes in wild type supplemented to a final concentration of 5 mM was set at 1, and values obtained with RNA isolated from *Δpsd1Δpsd2* supplemented with 5 mM or 0.25 mM ethanolamine, respectively, were set in relation. Data are mean values from three independent experiments with the respective deviation.

The question remained whether or not there was a direct correlation between the PE level and expression (transcription) levels of the three genes under closer investigation. To test this hypothesis, more stringent conditions for PE production in a double mutant deleted of *PSD1* and *PSD2* grown on minimal medium supplemented with 5 mM and 0.25 mM ethanolamine, respectively, was chosen. Under these conditions, the PE level in wild type (~ 16% PE of total phospholipids) dropped to ~8.5% PE in *∆psd1∆psd1* with the higher supplementation of ethanolamine, and to ~ 4.5 % PE with the lower supplementation of ethanolamine. As can be seen from Figure 3 the expression levels of *GPH1*, *GPM2* and *RSB1* do not strictly correlate with the depletion of PE in the cells. Whereas in *∆psd1∆psd2* supplemented with 5 mM ethanolamine the expression levels of all three genes were increased, although at a different extent, the expression levels of *GPH1* and *GPM2* dropped close to wild type when cells were only supplemented with 0.25 mM ethanolamine. In contrast, increasing levels of *RSB1* expression were observed with decreasing amounts of PE present in cells. In this case a correlation between changes in the amount of PE in the cell and the gene expression level may be given.

### Subcellular localization of Gph1p, Gpm2p and Rsb1p analyzed by fluorescence microscopy

**Figure 4:**
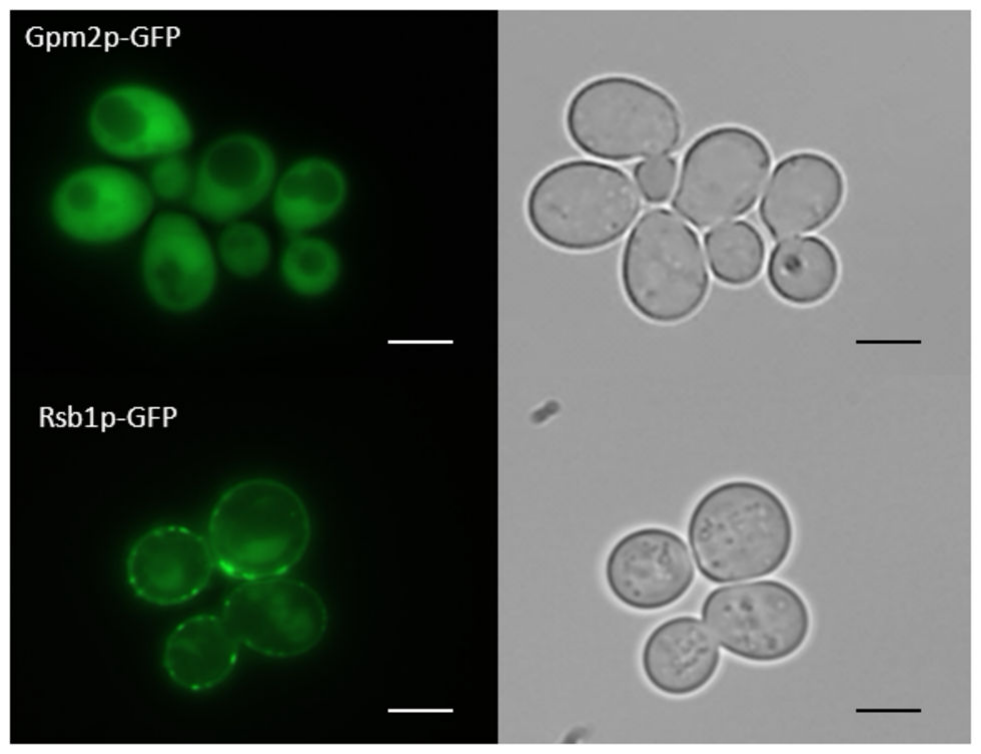
Subcellular localization of Gpm2p-GFP and Rsb1p-GFP by fluorescence microscopy. Fluorescence microscopy was carried out as described in the Methods section. Cells were grown in minimal medium –ura –met at 30°C to the late exponential phase and GFP fluorescence (left panel) was detected as described. Corresponding transmission microscopy of cells is shown in the right panel Subcellular distribution of Gpm2p-GFP (upper lane) and Rsb1p-GFP (lower lane) is shown. The size of the scale bar is 1 µm.

According to the SGD GO Slim Mapper (see Methods section), Gph1p, Gpm2p and Rsb1p are localized to the cytosol of yeast cells. This view was only partially confirmed in our fluorescence microscopy analysis of yeast strains bearing the respective GFP-tagged hybrids. Figure 4 demonstrates localization of GFP-Gpm2p throughout the cytoplasm. In contrast, GFP-Rsb1p was localized to the cell periphery in the form of distinct spots in or close to the plasma membrane. These spots may be specific domains of the plasma membrane or parts of the ER which are closely associated with the plasma membrane (plasma membrane associated membrane; PAM) [20].

**Figure 5:**
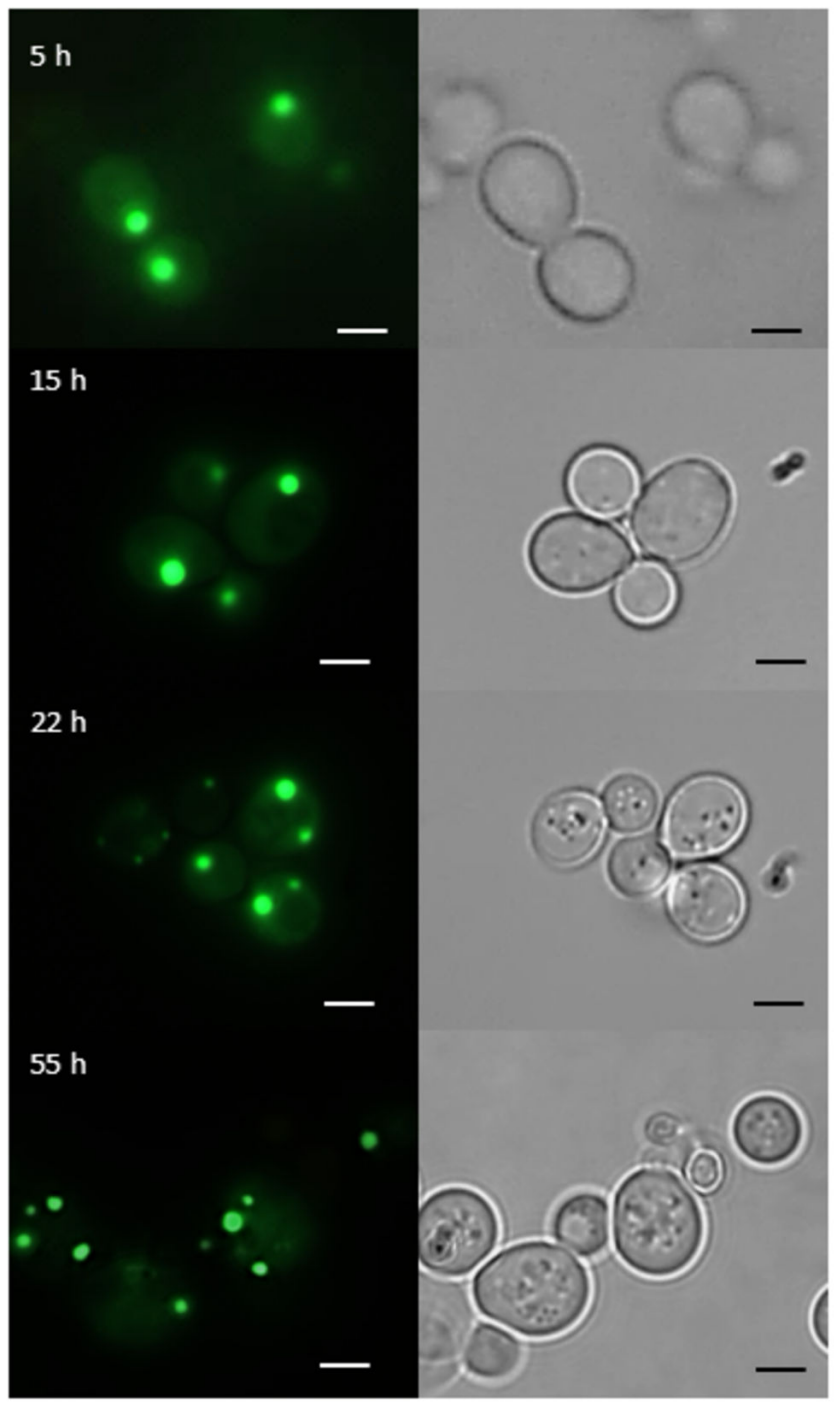
Subcellular localization of Gph1p-GFP by fluorescence microscopy. Fluorescence microscopy was performed as described in the legend to Figure 2. GFP fluorescence (left panel) and transmission microscopy (right panel) of cells bearing a Gph1p-GFP hybrid are shown. Cells were grown in minimal medium –ura –met at 30°C and pictures were taken from cells at early (5 h), middle (15 h) and late (22 h) exponentially phase and at the late stationary phase (55 h). The size of the scale bar is 1 µm.

Fluorescence microscopy revealed that GFP-Gph1p is localized to distinct spots in the cytoplasm (Figure 5). Gph1p is a glycogen phosphorylase, which degrades glycogen as carbohydrate source under conditions of nutrient limitation in the stationary phase and was reported to be associated with glycogen particles [39,40]. The present study confirms this localization of GFP-Gph1p. We also studied the subcellular distribution of GFP-Gph1p during various growth phases of the cell, namely in the lag phase (5 h), middle (15 h) and late (22 h) exponentially phase and late stationary phase (55 h). Whereas only single dots of the fluorescent signal were detected in cells during the early growth phases, older cells (55 h cultures) contained more of these particles. Additionally, diffuse fluorescence was observed throughout the cytoplasm, but not in the vacuole and in the nucleus. We excluded GFP-Gph1p accumulation in lipid droplets because staining of cells with Nile Red did not overlap with the signals of GFP-Gph1p (data not shown).

## Discussion

In the yeast, *Saccharomyces cerevisiae*, as in most other eukaryotic cells, the major membrane phospholipids are PC, PE, PI and PS. Phospholipid metabolism is governed by a complex network of reactions which are subject to strict genetic and biochemical regulation (for recent review see 41). ER and mitochondria are major sites of phospholipid synthesis [42] whereas other compartments such as the plasma membrane are devoid of phospholipid synthesizing enzymes [21]. Such membranes rely completely on the supply of lipids from other organelles.

The present study was focused on the global role of PE in the yeast and designed to study the genome wide response of *Saccharomyces cerevisiae* to PE depletion caused by deletion of *PSD1*. Psd1p is the major producer of PE in the yeast and localized to the inner mitochondrial membrane where it catalyzes decarboxylation of PS to PE [5–7]. For the present study a DNA-microarray analysis with a ∆*psd1* deletion mutant was performed. As described in the Results section, 54 genes were identified which were up-regulated in a ∆*psd1* deletion strain compared to wild type. The respective gene products serve several functions in diverse biological processes (see Tables 3-4). This large variety reflects the possible direct or indirect involvement of PE in many different cellular processes. As a matter of fact, DNA-microarray analysis provides information about genetic interactions, whereas functional links between reactions catalyzed by potentially interacting partner gene products are often hard to pinpoint. To narrow down the list of candidate genes which were detected in the DNA-microarray analysis we focused on growth phenotype and lipid analysis of mutants deleted of the identified genes. Surprisingly, only three mutants out of the collection of 54 candidates showed significant changes in growth phenotype and/or lipid profile, namely those affected in *GPM2*, *GPH1* and *RSB1*.

Gpm2p (glycerate phosphomutase 2) has been predicted as a non-functional homologue of Gpm1p which catalyzes the interconversion of 3-phosphoglycerate and 2-phosphoglycerate in the early glycolytic pathway of the yeast [43]. However, the fact that *GPM2* was subject to transcriptional regulation and the ∆*gpm2* deletion mutant exhibited growth defects on fermentable and non-fermentable carbon sources rather qualified *GPM2* as a functional gene. Despite these findings, a straight forward role of Gpm2p in phospholipids synthesis or regulation was not detected by our analyses. A slight growth defect of the ∆*gpm2* deletion mutant on lactate may explain a possible involvement of Gpm2p in respiration or energy metabolism, supported by some changes in the mitochondrial phospholipid pattern. The strong sensitivity to SDS (see Figure 2) of ∆*gpm2* and the altered levels of DMPE and PC in bulk membranes may be an indication for a link to membrane lipid metabolism and function.

Gph1p (glycogen phosphorylase 1) is localized to particles present in the cytoplasm (see Figure 5). This finding is in line with the physiological role of Gph1p which catalyzes the release of glucose 1-phosphate from glycogen and associates with glycogen particles [39,40]. In the stationary phase the distribution of GFP-tagged Gph1p was changing and the number of spots increased. This observation is most likely due to glycogen particle degradation under condition of starvation. Gph1p is known to be activated by a phosphorylase kinase and a cAMP dependent protein kinase, whereas glucose 6-phosphate, a product of glycolysis, inhibits Gph1p [39]. Therefore, Gph1p becomes only active when glucose 6-phosphate levels are decreasing. Whether or not these known functions of Gph1p are correlated with the novel observation that this protein is obviously also involved in the regulation of lipid metabolism (see Table 6) remains to be demonstrated. The growth defect of Δ*gph1* on non-fermentable carbon sources and in the presence of SDS (see Figure 2) combined with the changes of the lipid profile both in bulk membranes and especially in the plasma membrane (see Table 6) strongly supports this view. More detailed studies are currently in progress (Gsell et al., manuscript in preparation).

Among the short-listed candidate genes affected by *PSD1* deletion, *RSB1* is the only one with a direct link to lipid metabolism. It was suggested that Rsb1p (resistance to sphingoid long-chain base) is involved in lipid translocation across the plasma membrane [44–46]. It was suggested that Rsb1p may function as a transporter or flippase translocating LCBs (sphingoid long-chain bases) from the cytoplasmic side to the extracytoplasmic side of the plasma membrane [44]. Furthermore, it was found that the expression of Rsb1p was increased in cells with altered phospholipid asymmetry of the plasma membrane to compensate for compromised membrane functions by inappropriate distribution of LCBs between the inner and outer leaflet [47]. This finding may be linked to the observed up-regulation of *RSB1* under depletion of PE caused by *PSD1* deletion. Depletion of PE in Δ*psd1* causes changes in the phospholipid pattern of the plasma membrane. To compensate for possible defects due to these changes, expression of *RSB1* may be enhanced to maintain membrane stability. In a *∆rsb1* mutant the phospholipid pattern of the plasma membrane was not changed (see Table 6). However, moderate changes in the total cell extract and also in mitochondria such as decrease of PE and PC accompanied by an increase in PI might indicate an influence of Rsb1p on cellular lipid homeostasis.

In summary, our study identified a network of genes linked to function of *PSD1* in the yeast. Processes affected by depletion of PE through deletion of *PSD1* appear to wide spread (see Table 3-5) although defects may be secondary effects of changes in membrane behavior caused by PE depletion. At least in the cases of *GPM2*, *GPH1* and *RSB1*, a link to lipid metabolism and growth phenotype was established.

## Supporting Information

Table S1 **List of differentially expressed genes in Δpsd1 versus wild type.**
(DOCX)
