# Supplementary material for: Transcriptional Response to Deletion of the Phosphatidylserine Decarboxylase Psd1p in the Yeast Saccharomyces cerevisiae
Source: PLoS One. 2013 Oct 11;8(10):e77380. doi: 10.1371/journal.pone.0077380 (PMC3795641; doi:10.1371/journal.pone.0077380)
Supplement: Table S1 — List of differentially expressed genes in Δpsd1 versus wild type. (DOCX) [file pone.0077380.s001.docx]

Supplement Table S1

**List of differentially expressed genes in *Δpsd1* versus wild type**

| **Gene ID** | **Gene Symbols** | **Gene Title** | **logFC** | ***p* value** |
| --- | --- | --- | --- | --- |
| YNL169C | *PSD1* | Phosphatidylserine decarboxylase of the mitochondrial inner membrane, converts phosphatidylserine to phosphatidylethanolamine | -6,91 | 2,51E-11 |
| YLR327C | *TMA10* | Protein of unknown function that associates with ribosomes; putative homolog of the F1F0-ATPase synthase regulator Stf2p | 1,77 | 3,21E-05 |
| YEL011W | *GLC3* | Glycogen branching enzyme, involved in glycogen accumulation; green fluorescent protein (GFP)-fusion protein localizes to the cytoplasm in a punctate pattern | 1,07 | 0,00053906 |
| YBR047W | *FMP23* | Putative protein of unknown function; proposed to be involved in iron or copper homeostasis; the authentic, non-tagged protein is detected in highly purified mitochondria in high-throughput studies | 1,12 | 0,00071439 |
| YBR147W | *RTC2* | Putative vacuolar membrane transporter for cationic amino acids; likely contributes to amino acid homeostasis by exporting cationic amino acids from the vacuole; positive regulation by Lys14p suggests that lysine may be the primary substrate; member of the PQ-loop family, with seven transmembrane domains; similar to mammalian PQLC2 vacuolar transporter | 1,14 | 0,00072053 |
| YIL101C | *XBP1* | Transcriptional repressor that binds to promoter sequences of the cyclin genes, CYS3, and SMF2; expression is induced by stress or starvation during mitosis, and late in meiosis; member of the Swi4p/Mbp1p family; potential Cdc28p substrate | 2,09 | 0,0011918 |
| YDL169C | *UGX2* | Protein of unknown function, transcript accumulates in response to any combination of stress conditions | 1,16 | 0,00154061 |
| YMR251W | *GTO3* | Omega class glutathione transferase; putative cytosolic localization | 1,58 | 0,00154115 |
| YNL237W | *YTP1* | Probable type-III integral membrane protein of unknown function, has regions of similarity to mitochondrial electron transport proteins | 1,20 | 0,00156358 |
| YDL021W | *GPM2* | Homolog of Gpm1p phosphoglycerate mutase which converts 3-phosphoglycerate to 2-phosphoglycerate in glycolysis; may be non-functional derivative of a gene duplication event | 1,09 | 0,00168495 |
| YML100W | *TSL1* | Large subunit of trehalose 6-phosphate synthase (Tps1p)/phosphatase (Tps2p) complex, which converts uridine-5'-diphosphoglucose and glucose 6-phosphate to trehalose, similar to Tps3p and may share function; mutant has aneuploidy tolerance | 1,41 | 0,00179942 |
| YKR076W | *ECM4* | Omega class glutathione transferase; not essential; similar to Ygr154cp; green fluorescent protein (GFP)-fusion protein localizes to the cytoplasm | 1,01 | 0,00198524 |
| YOR185C | *GSP2* | GTP binding protein (mammalian Ranp homolog) involved in the maintenance of nuclear organization, RNA processing and transport; interacts with Kap121p, Kap123p and Pdr6p (karyophilin betas); Gsp1p homolog that is not required for viability | 1,01 | 0,00199361 |
| YAL061W | *BDH2* | Putative medium-chain alcohol dehydrogenase with similarity to BDH1; transcription induced by constitutively active *PDR1* and *PDR3* | 1,81 | 0,00210168 |
| YLR149C | *-* | Putative protein of unknown function; overexpression causes a cell cycle delay or arrest; null mutation results in a decrease in plasma membrane electron transport | 1,30 | 0,00224792 |
| YOR384W | *FRE5* | Putative ferric reductase with similarity to Fre2p; expression induced by low iron levels; the authentic, non-tagged protein is detected in highly purified mitochondria in high-throughput studies | 2,09 | 0,00251527 |
| YLR346C | *-* | Putative protein of unknown function found in mitochondria; expression is regulated by transcription factors involved in pleiotropic drug resistance, Pdr1p and Yrr1p; YLR346C is not an essential gene | 1,99 | 0,00255215 |
| YDR534C | *FIT1* | Mannoprotein that is incorporated into the cell wall via a glycosylphosphatidylinositol (GPI) anchor, involved in the retention of siderophore-iron in the cell wall | 1,72 | 0,00360081 |
| YHR087W | *RTC3* | Protein of unknown function involved in RNA metabolism; has structural similarity to SBDS, the human protein mutated in Shwachman-Diamond Syndrome (the yeast SBDS ortholog = *SDO1*); null mutation suppresses cdc13-1 temperature sensitivity | 1,58 | 0,00408903 |
| YGL121C | *GPG1* | Proposed gamma subunit of the heterotrimeric G protein that interacts with the receptor Gpr1p; involved in regulation of pseudohyphal growth; requires Gpb1p or Gpb2p to interact with Gpa2p; overproduction causes prion curing | 1,50 | 0,00470336 |
| YPR160W | *GPH1* | Non-essential glycogen phosphorylase required for the mobilization of glycogen, activity is regulated by cyclic AMP-mediated phosphorylation, expression is regulated by stress-response elements and by the HOG MAP kinase pathway | 1,28 | 0,00480749 |
| YNL194C | *-* | Integral membrane protein required for sporulation and plasma membrane sphingolipids content; has sequence similarity to *SUR7* and *FMP45*; GFP-fusion protein is induced in response to the DNA-damaging agent MMS | 1,18 | 0,00498808 |
| YJR005C-A | *-* | Putative protein of unknown function, originally identified as a syntenic homolog of an *Ashbya gossypii* gene | 2,46 | 0,00510371 |
| YOR289W | *-* | Putative protein of unknown function; transcription induced by the unfolded protein response; green fluorescent protein (GFP)-fusion protein localizes to both the cytoplasm and the nucleus | 1,15 | 0,0052983 |
| YKL071W | *-* | Putative protein of unknown function; expression induced in cells treated with the mycotoxin patulin, and also the quinone methide triterpene celastrol; green fluorescent protein (GFP)-fusion protein localizes to the cytoplasm | 1,00 | 0,00538264 |
| YHL047C | *ARN2* | Transporter, member of the ARN family of transporters that specifically recognize siderophore-iron chelates; responsible for uptake of iron bound to the siderophore triacetylfusarinine C | 1,51 | 0,00553745 |
| YFR053C | *HXK1* | Hexokinase isoenzyme 1, a cytosolic protein that catalyzes phosphorylation of glucose during glucose metabolism; expression is highest during growth on non-glucose carbon sources; glucose-induced repression involves the hexokinase Hxk2p | 1,22 | 0,00574348 |
| YJL105W | *SET4* | Protein of unknown function, contains a SET domain | 1,50 | 0,00588631 |
| YOR383C | *FIT3* | Mannoprotein that is incorporated into the cell wall via a glycosylphosphatidylinositol (GPI) anchor, involved in the retention of siderophore-iron in the cell wall | 1,11 | 0,00627485 |
| YOR049C | *RSB1* | Suppressor of sphingoid long chain base (LCB) sensitivity of an LCB-lyase mutation; putative integral membrane transporter or flippase that may transport LCBs from the cytoplasmic side toward the extracytoplasmic side of the membrane | 1,58 | 0,00642199 |
| YDR406W | *PDR15* | Plasma membrane ATP binding cassette (ABC) transporter, multidrug transporter and general stress response factor implicated in cellular detoxification; regulated by Pdr1p, Pdr3p and Pdr8p; promoter contains a PDR responsive element | 1,22 | 0,00642509 |
| YMR105C | *PGM2* | Phosphoglucomutase, catalyzes the conversion from glucose-1-phosphate to glucose-6-phosphate, which is a key step in hexose metabolism; functions as the acceptor for a Glc-phosphotransferase | 1,18 | 0,00662036 |
| YER037W | *PHM8* | Lysophosphatidic acid (LPA) phosphatase involved in LPA hydrolysis in response to phosphate starvation; phosphatase activity is soluble and Mg2+ dependent; expression is induced by low phosphate levels and by inactivation of Pho85p | 1,03 | 0,00663793 |
| YIR041W  YCR104W  YKL224C  YMR325W  YOR394W  YPL282C | *PAU15*  *PAU3*  *PAU16*  *PAU19*  *PAU21*  *PAU22* | Part of 23-member seripauperin multigene family encoded mainly in subtelomeric regions, active during alcoholic fermentation, regulated by anaerobiosis, negatively regulated by oxygen, repressed by heme /// Hypothetical protein /// Putative protein of unknown function | 1,59 | 0,00697615 |
| YGL156W | *AMS1* | Vacuolar alpha mannosidase, involved in free oligosaccharide (fOS) degradation; delivered to the vacuole in a novel pathway separate from the secretory pathway | 1,43 | 0,00717477 |
| YBR072W | *HSP26* | Small heat shock protein (sHSP) with chaperone activity; forms hollow, sphere-shaped oligomers that suppress unfolded proteins aggregation; oligomer activation requires a heat-induced conformational change; also has mRNA binding activity | 1,80 | 0,00788137 |
| YFL020C | *PAU5* | Member of the seripauperin multigene family encoded mainly in subtelomeric regions; induced during alcoholic fermentation; induced by low temperature and also by anaerobic conditions; negatively regulated by oxygen repressed by heme | 1,22 | 0,00831995 |
| YJL052W | *TDH1* | Glyceraldehyde-3-phosphate dehydrogenase, isozyme 1, involved in glycolysis and gluconeogenesis; tetramer that catalyzes the reaction of glyceraldehyde-3-phosphate to 1,3 bis-phosphoglycerate; detected in the cytoplasm and cell-wall | 1,06 | 0,00832004 |
| YHL040C | *ARN1* | Transporter, member of the ARN family of transporters that specifically recognize siderophore-iron chelates; responsible for uptake of iron bound to ferrirubin, ferrirhodin, and related siderophores | 1,05 | 0,00885204 |
| YHR096C | *HXT5* | Hexose transporter with moderate affinity for glucose, induced in the presence of non-fermentable carbon sources, induced by a decrease in growth rate, contains an extended N-terminal domain relative to other HXTs | 1,74 | 0,00899573 |
| YGR066C | *-* | Putative protein of unknown function | 1,57 | 0,00974168 |
| YOL052C-A | *DDR2* | Multistress response protein, expression is activated by a variety of xenobiotic agents and environmental or physiological stresses; also known as: DDRA2 | 1,56 | 0,01090044 |
| YGR248W | *SOL4* | 6-phosphogluconolactonase with similarity to Sol3p | 1,14 | 0,01197417 |
| YJR150C | *DAN1* | Cell wall mannoprotein with similarity to Tir1p, Tir2p, Tir3p, and Tir4p; expressed under anaerobic conditions, completely repressed during aerobic growth | 2,72 | 0,01355517 |
| YOR382W | *FIT2* | Mannoprotein that is incorporated into the cell wall via a glycosylphosphatidylinositol (GPI) anchor, involved in the retention of siderophore-iron in the cell wall | 1,34 | 0,01460368 |
| YMR317W | *-* | Putative protein of unknown function with some similarity to sialidase from Trypanosoma | 1,33 | 0,01474489 |
| YFL014W | *HSP12* | Plasma membrane protein involved in maintaining membrane organization in stress conditions; induced by heat shock, oxidative stress, osmostress, stationary phase, glucose depletion, oleate and alcohol; regulated by HOG and Ras-Pka pathways | 2,05 | 0,01889002 |
| YOR009W | *TIR4* | Cell wall mannoprotein of the Srp1p/Tip1p family of serine-alanine-rich proteins; expressed under anaerobic conditions and required for anaerobic growth; transcription is also induced by cold shock | 1,18 | 0,03393083 |
| YOR237W | *HES1* | Protein implicated in the regulation of ergosterol biosynthesis; one of a seven member gene family with a common essential function and non-essential unique functions; similar to human oxysterol binding protein (OSBP) | 1,07 | 0,03526453 |
| YLR136C | *TIS11* | mRNA-binding protein expressed during iron starvation; binds to a sequence element in the 3'-untranslated regions of specific mRNAs to mediate their degradation; involved in iron homeostasis | 1,27 | 0,04999745 |
